# Anti-inflammatory topical medication – new developments in the treatment of atopic dermatitis 

**DOI:** 10.5414/ALX02255E

**Published:** 2021-08-27

**Authors:** Johannes Wohlrab, Burkhard Kreft, Luisa Sophie Scholz

**Affiliations:** 1 University Hospital Halle (Saale), Clinic for Dermatology and Venereology, and; 2 Institute for Applied Dermatopharmacy, Martin Luther University Halle-Wittenberg, Halle (Saale), Germany

**Keywords:** atopic dermatitis, inflammation, topical therapy, topical medication

## Abstract

Atopic dermatitis is a chronic inflammatory disease that arises from polygenic disposition, a dysfunction of the physicochemical epithelial barrier, a cutaneous dysbiosis, and a faulty neurosensory activity and shows a highly individual acuity due to epigenetic factors. An essential component of therapeutic management is the application of anti-inflammatory topical medication. Currently, topical glucocorticoids and topical calcineurin inhibitors are routinely used in reactive and proactive therapy. In recent years, the development of molecular medicine has identified several new therapeutic targets that have enabled the development of innovative therapeutic approaches. In addition to phosphodiesterase-4 inhibitors and aryl hydrocarbon receptor modulators, it is mainly Janus kinase inhibitors with different selectivity that are emerging as new effective and safe options for topical therapy. The current data suggests that in the coming months and years representatives of the above-mentioned substance classes will be approved for topical use.

## Background 

**Figure 1:**
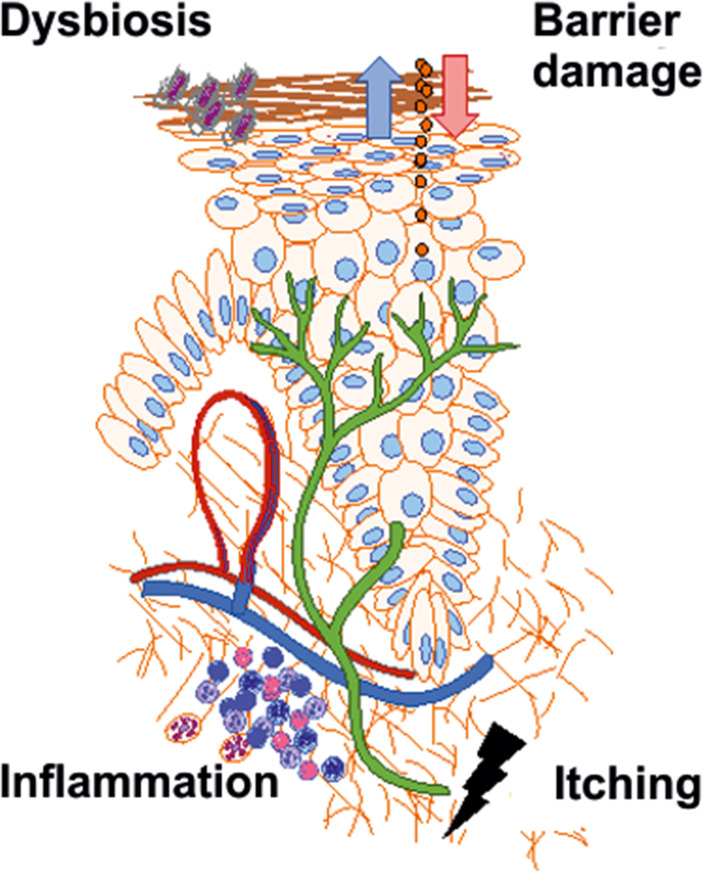
Schematic representation of the pathogenetic factors of atopic dermatitis.

Atopic dermatitis (AD) represents a disease of type 2 immunity as well as a complex dysregulation of the physicochemical and microbial barrier of the stratum corneum. Based on a polygenic disposition, facilitated by the barrier damage, after exogenous triggering for example by allergens, pollutants, microbial antigens, toxins, or scratching (so-called exposome), interleukin 25 (IL-25), IL-33 as well as thymic stromal lymphopoietin (TSLP) are released as so-called “alarmins” by keratinocytes [1]. These condition the activation of dendritic cells (DC) as well as the release of IL-13 by innate lymphoid cells 2 (ILC2). The antigen-presenting DCs migrate to regional lymph nodes and activate antigen-specific naive CD4+ T cells, which then differentiate into type 2 effector T helper cells (Th2) and form various Th2 subtypes (subsets) [1]. IL-13 also activates tissue eosinophil migration and B cells, in which increased IgE formation (Ig-class switch) is induced. DCs and B cells cause the release of IL-4 and IL-13, which are considered master cytokines of AD and are at the center of inflammatory pathogenesis. Closely related to the inflammatory event, IL-31 causes the activation of neurosensory afferents, which is essential for the development of chronic pruritus [2] (Figure 1). 

Comparable molecular cascades and immunological processes can also be demonstrated in the epithelia of the nasal mucosa, lung, and intestine. This pattern of inflammatory pathogenesis of AD highlights that the inflammatory acuity of the disease is strongly influenced by interindividually varying genetic predisposition and epigenetic influence through the “exposome.” Frequently, clinically chronic disease phases with interpolation of acute inflammatory relapses appear in childhood and adolescence or lifelong in phenotypically varying emphasis of individual body areas. With increasing disease duration and severity, the frequency of the development of comorbidity also increases. Depending on the individual course of the disease and the clinical need for treatment derived from the acute nature, a topical agent alone or in combination with systemic therapy is recommended for anti-inflammatory treatment. Only in the phase of maintenance therapy with an efficient systemic therapeutic agent is it possible in most cases to omit the use of anti-inflammatory topical medication. Against this background and considering a lifetime prevalence of AD of 14.3% as well as the severity distribution in Germany (PO-SCORAD: mild 26%, moderate 57%, severe 17%), the importance of anti-inflammatory topical therapy becomes clear [3]. 

## Anti-inflammatory topical medication 

The use of topical medication with anti-inflammatory agents is propagated in a phase-specific manner. This means that the choice of vehicle system and the active drug ingredient are selected and adjusted according to the disease activity in the therapeutic area [4]. This selection has a direct impact on the patient’s tolerability and adherence to topical therapy [5]. Essentially, active ingredients from two major drug classes, glucocorticoids and calcineurin inhibitors, are used. The use of historical ingredients, such as tar or zinc oxide in specialty or magistral formulations, is no longer of practical relevance today. In contrast, both topical glucocorticoids (TGCs) and topical calcineurin inhibitors (TCIs) are substance classes validated in their benefit-risk profile and are effective and safe when used properly [6]. Among the TCIs, active substances of different potency (according to Niedner 1 – 4) and with a different benefit-risk-profile (benefit-risk ratio) are distinguished according to the therapeutic index (TIX) [7, 8]. For the experienced therapist, this results in a comprehensive armamentarium for professional use. If used appropriately and taking into account the dose-response relationship, also for adverse effects, concerns or even a cortisone phobia are unfounded. However, everyday practice teaches that, unfortunately, the pharmacological relationships are all too often disregarded, especially through overly long-term and uncritical use of TGCs. In contrast, the use of TCI has proven to be particularly effective in the maintenance phase of therapy and in long-term use. Here, with tacrolimus and pimecrolimus, two active ingredients with different potencies are available which, despite their high lipophilicity and large molar mass, allow variability in clinical practice due to their formulation in different galenic systems. For the topical therapy of atopic conjunctivitis, ciclosporin-containing eye drops are also available as a specialty or extemporaneous formulation. Both classes of substances can be used in both reactive and proactive therapy of the disease [9, 10]. 

In recent years, advances in molecular medicine have identified new targets for therapeutic intervention in AD. At the same time, developments in biotechnology enabled the application of recombinant proteins, fusion proteins, and monoclonal antibodies to target therapy in systems therapeutics [11]. Biotechnology does not stop at topical application either, although the hurdles for application are disproportionately higher here due to the high molecular masses of the active ingredients as well as stability problems. Nevertheless, active ingredients for specific applications such as ncRNA or antagomirs are also being developed here and are referred to as “BioTopicals” [12]. Of particular interest, however, are small-molecule agents that can be made bioavailable in the skin using common galenic systems. 

### Phosphodiesterase-4 inhibitors 

Phosphodiesterase-4 (PDE4) inhibition for AD therapy has been discussed for many years. The anti-inflammatory efficacy of PDE4 inhibitors (PDE4i) can be explained by broad, nonselective inhibition of proinflammatory cytokines, inhibition of DCs, T cells, and transcription factors, and effects at the T cell receptor. The approval of apremilast in the anti-inflammatory systemic therapy of psoriasis has given new activity to these efforts. Outside the EU, an approval of a crisaborole preparation (Eucirsa^®^ 2% Ointment, Pfizer, Mission KS, USA) for the treatment of AD has existed for several years, but without relevant advantage over TCI [13, 14]. Comparable efficacy data are also shown by E6005/RVT-501, a topical PDE4i tested in Japan, and LEO 29102 in earlier study phases. Currently, a 0.05% roflumilast (ARQ-151) cream is being tested in a phase 3 trial in children. In adults, significantly higher doses of 0.15% have not met the primary endpoint. However, a sustained assessment is not possible here because not all study data have been published. Phase 2 data on topical use of difamilast (OPA-15406) in an ointment preparation showed comparable good data in dose finding for 0.3% and 1.0% with a significant difference from vehicle [15]. Again, there are still too few data to evaluate the potency. Overall, however, it is clear that PDE4 inhibition is an effective concept for anti-inflammatory therapy of AD. 

### Janus kinase inhibitors 

The use of Janus kinase inhibitors (JAKi) is considered to have great potential not only in systemic but also in topical application for anti-inflammatory therapy of AD. The JAK/STAT signal transduction pathway is evolutionarily ancient and important for basal regulatory processes of the cell as well as equally important for defense against microbial noxious agents, inflammation, and neuroimmunological processes. It is a cellular pathway of signal transduction that leads to ligand-induced activation of transmembrane receptor-associated JAKs, which, via phosphorylation of the intracellular portions, results in the availability of various signal transducers and activators of transcription (STATs). These enter the nucleus and regulate gene expression by direct binding to DNA. Different JAKs (JAK1-3, TYK2) and their selective binding to different receptor chains activate different STATs (STAT1-6) and thereby induce different gene expression patterns. In this process, the JAKs can occur in different hetero- or homodimers [16]. According to current knowledge, JAK1 and JAK2 inhibition are of primary importance for the therapeutic effect in AD. At the same time, the role of another kinase of tyrosine-protein kinase (SYK) is discussed. The different JAKi have different selectivities with respect to the inhibition of the individual JAKs, based on the IC50 values of the active substances. All JAKi are small-molecule substances that are basically suitable for topical application; however, some of them differ considerably in half-life, protein binding as well as elimination behavior. Data for topical application in the indication AD are available for tofacitinib (pan-JAKi), ruxolitinib (JAK1/2i) as well as delgocitinib (JAK1/2/3/TYK2i) and cerdulatinib (JAK1/2/3/TYK2/SYKi). Efficacy in AD has been demonstrated for all of the above agents in early studies. Only for a 0.5% delgocitinib ointment preparation phase 3 data are available so far, which showed a decrease from baseline for the mEASI score of 44.3% in the active-agent group and –1.7% in the vehicle group. In Japan, the preparation is already approved for topical therapy of AD under the trade name Corectim^®^ 0.25 and 0,5% ointment (Tobacco Inc., Tokyo, Japan) [17]. 

### Aryl hydrocarbon receptor modulators 

In recent years, the importance of the aryl hydrocarbon receptor (AhR) has been highlighted. This is a cytoplasmic receptor system that migrates to the nucleus after complexation with appropriate ligands and here induces the expression of anti-inflammatory cytokines as well as barrier proteins (for example, filaggrin) via the regulation of transcription factors. In this context, AhR was identified as an essential target of historical tar treatment so that ligands of the receptor are also called “tar smarts”. The first compound developed in this context as a ligand of AhR is tapinarof (GSK2894512, or benvitimod). In a 1.0% cream preparation, tapinarof was initially investigated in the indication psoriasis. The efficacy and good tolerability derived from the results of early study phases were then also tested in AD. Here, a 0.5% preparation was used, and a difference in efficacy compared to the vehicle was shown after 12 weeks with once and twice daily application. However, these phase 2b data do not yet allow an evaluation [18]. However, these results show that AhR is a therapeutic target of practical relevance. 

### Cannabidiol 

The importance of the cannabinoid receptor system (CB1, CB2) of the skin in the context of neurosensory activity of afferents (pruritus), but also in the regulation of inflammatory processes, has long been known. The use of cannabis extracts in topical medications is not purposeful and simply prohibited due to the content of tetrahydrocannabinol (THC) and the psychoactive effects associated with it, even after transdermal application. When cannabidiol (CBD) is used, the risk of psychoactive effects does not exist or does not exist to a relevant degree. Nevertheless, CBD is not uncritically viewed pharmacologically, since anticonvulsive, neuroprotective, and antioxidant effects are mediated via central nervous receptors. CBD is even marketed as a food supplement and food additive in many countries. However, the German Federal Office of Consumer Protection and Food Safety criticizes the marketability of CBD oils, which are also available in Germany, as they are neither approved as medicinal products nor as food (novel food). However, CBD with a THC content of less than 0.2% does not fall under the German Narcotics Act. However, the responsible EU Commission has classified CBD as a narcotic so that there is a contradictory legal situation here, which currently stands in the way of the development of topical medications containing CBD. However, a study was recently published in the USA investigating the use of a 1.0% CBD-containing gel in AD. This proof-of-concept study showed not only a decrease in pruritus but also a reduction in EASI score [19]. Hopefully, pharmaceutical development of CBD-containing topical medications for use in AD outside of narcotic status will become feasible, and larger studies can provide objective verification of efficacy. 

## Conclusion 

It is clear from the above that various groups of compounds have great potential for use in topical medication. Due to the extensive developments and the already almost confusing study situation, it seems very likely that especially topical JAKi and possibly PDE4i or tapinarof as AhR ligand will reach market maturity and receive approval in the near future. It remains to be seen, however, whether new risks of topical therapy may arise. Especially for JAKi, however, there is currently no evidence for this [20]. In addition to new active substances, innovative galenic systems with known and new active substances are also being developed, thus further enriching the therapeutic options in AD. Furthermore, new developments for barrier-protective and antipruritic therapy as well as for the regulation of dysbiosis are also emerging so that overall a multitude of options for a holistic, individualized management of the complex disease AD can be expected. 

## Funding 

None. 

## Conflict of interest 

J.W. discloses having received grants for scientific projects, clinical trials, lectures, or consulting in the last 5 years from the following companies: Abbvie, Actelion, Allergika, Almirall, Aristo, BayPharma, Beiersdorf, Biogen, Boehringer, Dermapharm, Evolva, Galderma, GSK, Helm, Hexal, Infectopharm, Janssen-Cilag, Jenapharm, Johnson & Johnson, Leo, Lilly, L’Oréal, Medac, Medice, Mibe, MSD, Mylan, Novartis, Pierre Fabre, Pfizer, Riemser, Skinomics, ViiV, Wolff. 

B.K. and L.S.S. do not declare any conflicts of interest.
